# Supplementary material for: Prothrombin prevents fatal T cell–dependent anemia during chronic virus infection of mice
Source: JCI Insight. 2025 Jan 16;10(4):e181063. doi: 10.1172/jci.insight.181063 (PMC11949038; doi:10.1172/jci.insight.181063)
Supplement: Supplemental data [file jciinsight-10-181063-s189.pdf]

SUPPLEMENT

Prothrombin prevents fatal T cell-dependent anemia during chronic virus infection of mice

Rachel Cantrell*, H. Alex Feldman*, Leah Rosenfeldt, Ayad Ali, Benjamin Gourley, Cassandra Sprague, Daniel Leino, Jeff Crosby, Alexey Revenko, Brett Monia, Stephen N. Waggoner#, Joseph S. Palumbo#

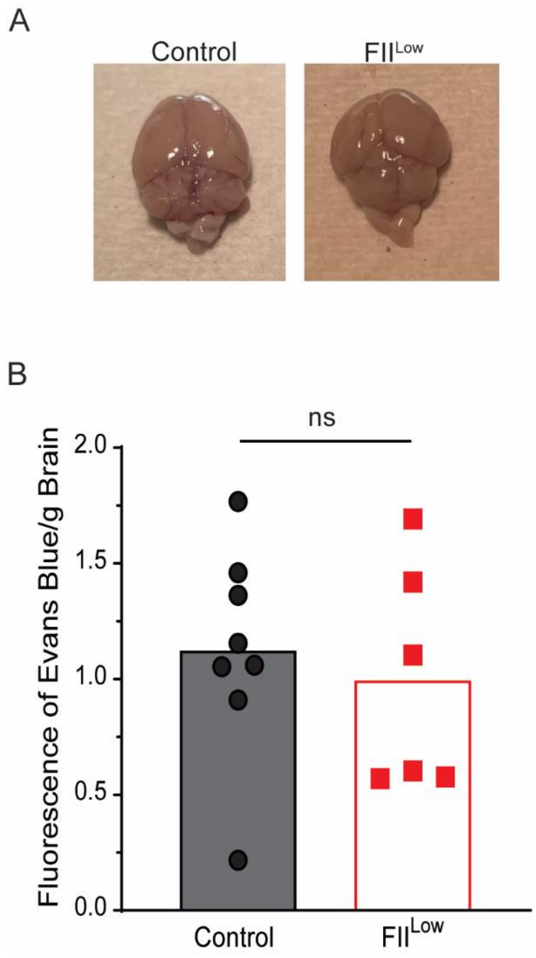

Supplemental Figure 1. No evidence for vascular leakage in low prothrombin infected mice. C57BL/6 mice underwent pharmacologic depletion of prothrombin (FII^{Low}, red squares) or control treatment (black circles) followed by intravenous infection with $2x10^6$ PFU of clone 13 LCMV (n=6-8 mice/group). At day 6 of infection, Evan's blue dye was administered intravenously two hours prior to euthanasia. Brains were harvested and analyzed (**A**) visually as well as (**B**) quantitatively. ns: $p>0.05$ by unpaired Student's t-test.

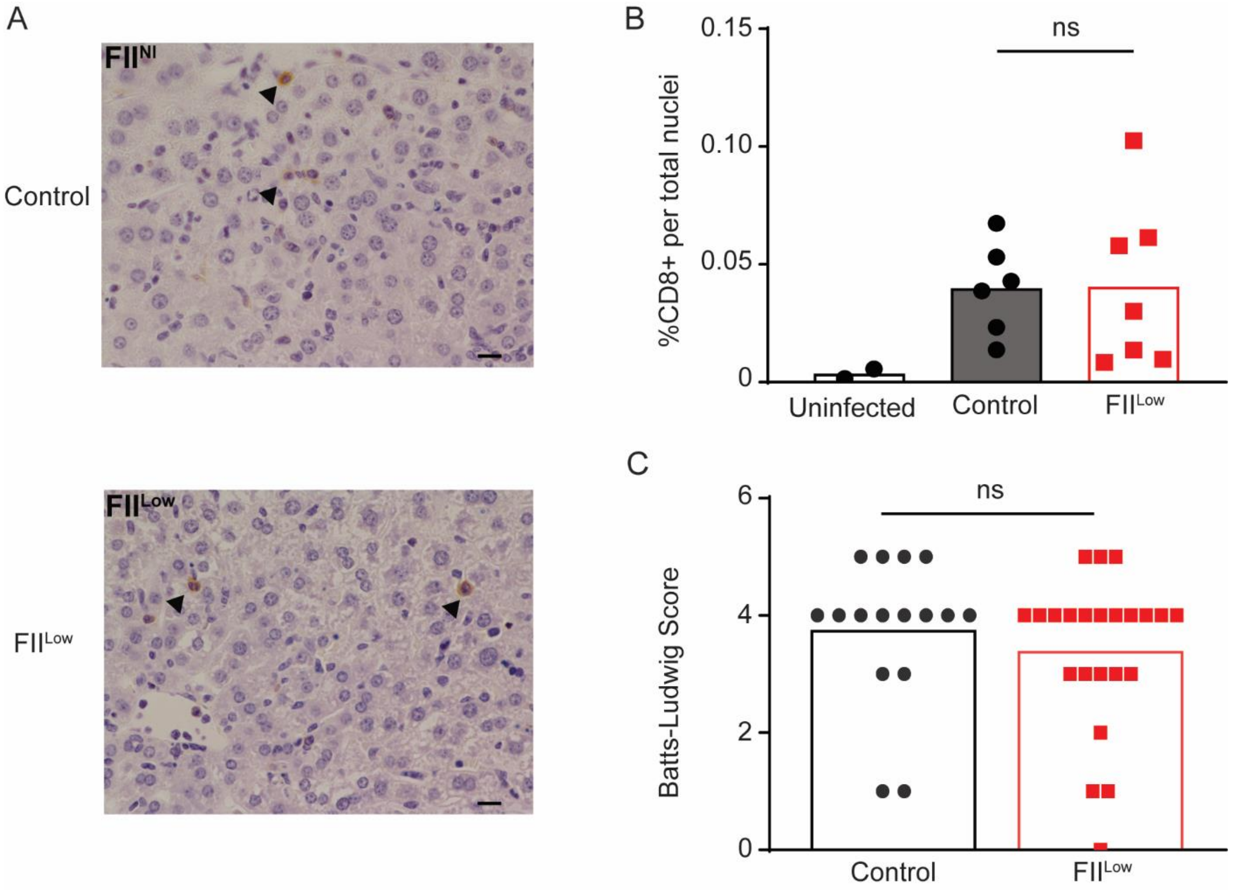

Supplemental Figure 2. No enhanced liver infiltration of CD8+ T cells in low prothrombin infected setting. C57BL/6 mice underwent pharmacologic depletion of prothrombin (FII^{Low}, red squares) or control treatment (black circles) followed by intravenous infection with $2x10^6$ PFU of clone 13 LCMV (n=6-7/group, and 2 uninfected controls). At day 8 of infection, livers were harvested and stained for CD8α. (**A**) Representative images and (**B**) quantification are shown. (**C**) These livers sections and subjected to hematoxylin and eosin prior to blinded Batts-Ludwig scoring by a pathologist. Livers from uninfected mice (n=2) scored ≤1 in this assessment. ns: p>0.05 by unpaired Student's t-test.

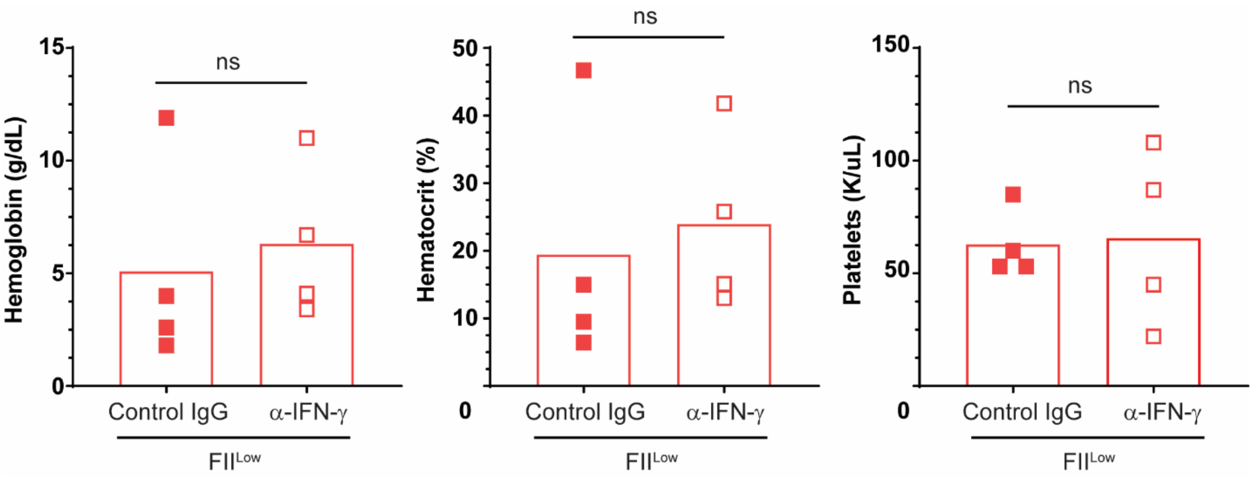

Supplemental Figure 3. IFN-γ neutralization does not rescue anemia in infected low prothrombin mice. C57BL/6 mice underwent pharmacologic depletion of prothrombin (FII^{Low}) followed by intravenous infection with $2x10^6$ PFU of clone 13 LCMV. On days 2 and 5 of infection, mice received retroorbital infections of 200 µg anti-mouse IFN-γ (α-IFN-γ, open red squares) or control IgG1 antibody (Control IgG, closed red squares, n=4/group). On day 8, blood was analyzed for hemoglobin levels, hematocrit, and platelet counts. ns refers to p>0.05 by unpaired Student's t-test.

A

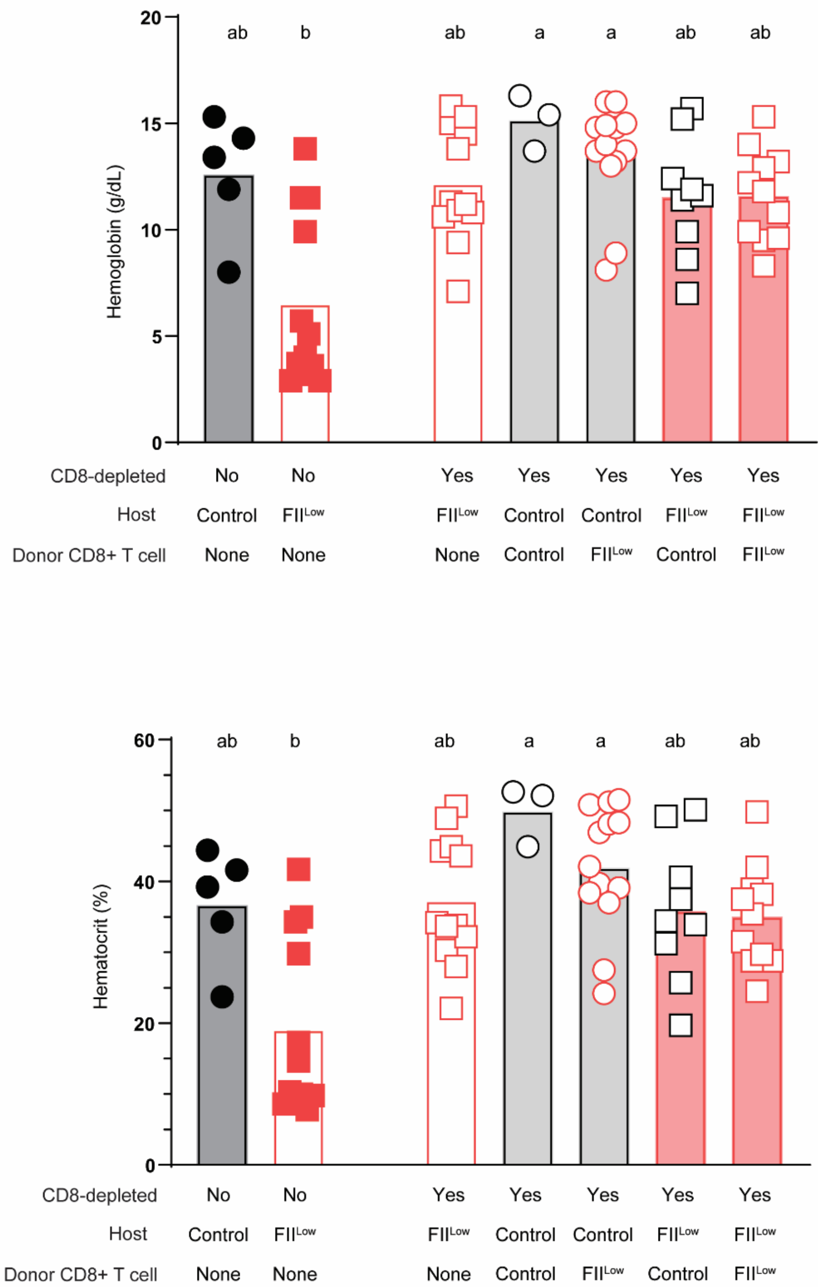

B

Supplemental Figure 4. IFN-γ neutralization does not rescue anemia in infected low prothrombin mice. C57BL/6 mice underwent pharmacologic depletion of prothrombin (FII^{Low},) or control treatment. Some mice then received anti-CD8 antibodies (noted in top row as YES) to deplete CD8+ T cells or isotype control antibody (Noted in top row as No) one day prior to intravenous infection with $2x10^6$ PFU of clone 13 LCMV. Donor mouse spleen CD8+ T cells were isolated on day 6 and 100,000 of these cells (third denotes donor mouse source of CD8+ T cells) were injected into groups of host mice (treatment listed on 2nd row) on day 4 of infection. On day 8, recipient mouse blood was analyzed for (**A**) Hemoglobin and (**B**) hematocrit. Means followed by a common letter are not significantly different by ordinary one-way ANOVA, followed by Tukey's multiple comparisons test, with single pooled variance.
